# Association between serum osteocalcin and atherosclerosis in Type-2 diabetes mellitus: a cross-sectional study

**DOI:** 10.1186/s12902-023-01462-8

**Published:** 2023-12-06

**Authors:** Vishal Chandra Sharma, Sudha Vidyasagar, Cynthia Amrutha Sukumar, Nanda Krishna B, Sharanya Shree

**Affiliations:** 1 Department of Medicine, Kasturba Medical College, Manipal, Manipal Academy of Higher Education, Manipal, Karnataka India; 2 Department of Medicine, Trinity Health, Oakland, CA USA

**Keywords:** Osteocalcin, Carotid intima thickness, atherosclerosis, CC-IMT, Type 2 diabetes mellitus, inflammation

## Abstract

**Background:**

The past few decades have seen a marked increase in the macrovascular complications of Type-2 diabetes mellitus (T2DM) such as coronary heart disease, peripheral arterial disease, and cerebrovascular disease. This has been predominantly attributed to the increased atherosclerosis in these patients. Atherosclerosis usually remains an asymptomatic condition and this poses a significant challenge in its early diagnosis and timely intervention. Hence, there is an immediate need for exploring novel tools to aid in the early detection of atherosclerosis, especially in T2DM patients. Osteocalcin (OC), synthesized by osteoblasts, is a protein hormone found in the skeletal system. This protein is considered as a marker for bone density and in recent times has been gaining interest due to its protective role in cerebrovascular diseases(CVD).

**Methods:**

We conducted a cross-sectional study and evaluated the association between serum OC levels and atherosclerosis in 113 T2DM patients. Carotid intima-media thickness (CC-IMT) was used as an estimate of atherosclerosis and patients were divided into two groups (CC-IMT < 0.9 and ≥ 0.9). Correlation of serum OC levels and glycemic parameters and lipid profiles were studied and compared between both groups.

**Results:**

There is a significant negative correlation between the CC-IMT estimates and serum OC levels. CC-IMT also has a significant association with other biochemical parameters such as fasting blood sugar, glycated hemoglobin and high-density lipoprotein.

**Conclusion:**

Although the independent association of serum OC could not be established in the T2DM patient population, overall, the results favor low serum OC as a prognostic marker for atherosclerosis.

## Introduction

Atherosclerosis remains the main cause of death and morbidity in the world and imposes a significant burden especially on the Indian population [1–3]. India is the diabetic capital of the world and diabetes mellitus is an established risk factor for atherosclerosis [4, 5]. It is postulated that in diabetic patients, the high glucose levels have a pro-inflammatory effect on endothelial cells. Theadvanced glycation end-products (AGEs) or reactive oxygen species of the vessels contribute to atherosclerosis. Many diabetic patients are also associated with metabolic syndrome which further increases the risk of developing atherosclerosis. Hence early detection and treatment of atherosclerosis in diabetic patients play a pivotal role in preventing further vascular complications. In this regard, novel biomarkers are being studied to understand the disease dynamics of atherosclerosis to help in early detection and management in patients with type-2 diabetes mellitus (T2DM). Osteocalcin (OC) is one such molecule found to have a link in the pathogenesis of atherosclerosis by its effect on endothelium and its effects on glucose metabolism.

OC, also known as the bone gamma-carboxy glutamic acid-containing protein (BGLAP), synthesized by osteoblasts, is a small (49-amino-acid) non-collagenous protein hormone found in the skeletal system [6]. This protein is considered as a marker for bone density variations and in recent times has been gaining interest due to its protective role in CVD. It is proposed that OC can retard vascular endothelial dysfunction through this insulin-sensitizing effect [7].

To determine the extent of severity of atherosclerosis and the associated risks, a non-invasive technique of measurement of carotid artery intima-media thickness (IMT) using high-resolution B-mode ultrasonography is often used [8–10].

Studies have shown conflicting results regarding the association between atherosclerosis and OC. A cross-sectional study done on 72 T2DM patients in Spain concluded that OC was associated with atherosclerosis [11]. A large meta-analysis and systematic review also noted that the presence of OC-positive cells had a consistent positive correlation with atherosclerosis [12]. A Russian study evaluated the association between OC and atherosclerosis and found significantly higher concentration of OC in atherosclerotic calcified plaques [13].

Minfang Zhang et al. conducted a study in Shanghai, China to look for a relationship between OC levels and atherosclerosis in 240 patients of chronic kidney disease (CKD). They concluded that levels of Undercarboxylated OC (uOC) were significantly lower in patients CKD than in healthy individuals [14].

In a review by Zhang et al., it was found that uOC also showed a negative correlation with IMT. Another study found an inverse relationship between OC and CC-IMT and concluded that serum OC levels strengthen identifying subclinical (SC) atherosclerosis over ASCVD risk score, especially among patients with a moderate-high risk score [14].

Although the relationship between OC and atherosclerosis parameters in humans has been explored in recent studies, it has conflicting results and warrants further evaluation [11].

Our study has tried to establish a correlation between OC levels and atherosclerosis estimated by CC-IMT in T2DM patients. We compared serum OC with CC-IMT, and in those patients with macrovascular (MV) complications. We studied other risk factors for atherosclerosis like diabetic parameters like fasting blood sugar, glycated hemoglobin and parameters of lipid metabolism.

## Materials and methods

Study Design: A cross-sectional study was conducted in a tertiary care hospital in South India from September 2018 to 2020 on 113 diabetic adults. The inclusion criteria was patients with T2DM* who were above the age of 18 years. We excluded patients with type-1 DM, history of bone disorders such as Paget’s disorders, osteomalacia, thyroid and parathyroid disorders, acromegaly and growth hormone deficiency, chronic kidney diseases, chronic liver diseases, pregnant women, women currently using oral contraceptives, patients on vitamin K, calcium, calcitonin, biphosphates, vitamin-D supplements and any hormone-related therapy or steroids.

* *T2DM patients were screened according to the ADA (American Diabetes Association) criteria, which included Hemoglobin A1C ≥ 6.5%; Fasting plasma glucose (FPG) ≥ 126 mg/dL. 2-hour plasma glucose (PG) ≥ 200 mg/dL during oral glucose tolerance test; Random plasma glucose (PG) ≥ 200 mg/dL in persons with symptoms of hyperglycemia or hyperglycemic crisis.*

### Methodology

Data was collected as a response to a standard questionnaire. It included recorded demographics, and details including comorbidities, risk factors, and complications of atherosclerosis. All patients underwent a detailed structured physical examination that included evaluation of general and systemic parameters. Blood pressure measurements were made in all the included patients. A blood sample for assessing OC, fasting lipid profile, fasting plasma glucose, glycated hemoglobin was collected and processed. A blood sample was collected by venipuncture from peripheral veins under aseptic conditions. The first sample was collected after an overnight 8–10 h of fasting in the morning for baseline measurement of fasting plasma glucose, OC, glycated Hb, fasting lipid profile. Glucose was estimated by the hexokinase method. HbA1C was measured by High-Performance Liquid Chromatography method (HPLC) ion exchange-based method. Lipid profile estimation included TC (CE-CHOD-POD); TG (GPO Tinder); HDL (Direct-homogenous); LDL (Calculated). Renal function tests were done for urea (urease GLDH) and creatinine levels.

For OC, blood was allowed to clot, and serum was separated by centrifugation at room temperature and stored at -70 °C before the estimation of OC levels.

Carotid artery intima-media thickness was estimated using a B-Mode Ultrasound measurement performed by a qualified Radiologist.

Patients with CCIMT > 0.9 mm have been taken as those having atherosclerosis and with CCIMT < 0.9 mm as normal patients [15].

Serum OC levels were estimated using an ELISA test kit (DIAsource hOST-EASIA Kit from ‘DIAsource Immunoassays S.A.Rue du Bosquet, 2, B-1348 Louvain-la-Neuve, Belgium’) by following the manufacturer’s instructions. Serum OC levels were calculated from a final absorbance reading at 450 nm with a reference filter set at 630 nm (or 650 nm).

### Data analysis

The statistical analysis of the data was done by using SPSS software version 20.0 and GraphPad Prism software. The statistical test for means and proportions was performed using Student’s -test and Chi-square test, respectively. The statistical test for medians with interquartile range for parameters with skewed distribution was estimated using the Mann-Whitney U test. Using Levene’s test, we found that the data was normally distributed. The statistical level of significance was set at 5%. A correlation coefficient was used to determine any association between OC and atherosclerosis.

## Results

### Baseline biochemical data

A total number of 113 adults with T2DM with a mean age of 59 ± 12 years were included in the study. All the included patients underwent biochemical tests including measurement of serum OC. Demographics of the included participants and baseline biochemical parameters including fasting blood sugar (FBS) and HbA1C, and fasting lipid profile are presented in Tables 1 and 2, respectively.

**Table 1:** Baseline demographics of the study subjects

	Total N = 113	CCIMT < 0.9 mm N = 45(40%)	CCIMT ≥ 0.9 mm N = 68 (60%)	P-value
Age (years)				
Mean ± SD Min Max	59 ± 11.7 years 33 years 86 years	58.2 ± 11.2 years 36 years 86 years	60 ± 12.1 years 33 years 83 years	0.5302
Gender	N (%)			0.5647
Male	55 (49%)	20 (44%)	35 (51%)	
Female	58 (51%)	25 (56%)	33 (49%)	
Smoking				
Smoker Non- smokers	18 (16%) 95 (84%)	7 (16%) 38 (84%)	11 (16%) 57 (84%)	> 0.999
Duration of diabetes (years) mean, IQR Min Max	6 (4–9) 1 years 25 years	5 (3-7.5) 1 years 21 years	6 (4–9) 1 years 25 years	0.1346
Atherosclerotic disorders				
Present Absent	68 (60%) 45 (40%)	14 (31%) 31 (69%)	68 (100%)	
CC-IMT				
Mean ± SD	0.88 ± 0.36	0.55 ± 0.21	1.1 ± 0.25	< 0.0001^****^
Creatinine (mg/dl)				
Mean ± SD	0.856 ± 0.184	0.856 ± 0.168	0.856 ± 0.196	0.6141

***, **** represents statistical significance with a P-value of < 0.0001 and < 0.0001, respectively

**Table 2:** Biochemical parameters of the study population

	Total	CCIMT < 0.9 mm	CCIMT ≥ 0.9 mm	P-value
BIOCHEMICAL PARAMETERS	N = 113 Median (IQR)	N = 45 Median (IQR)	N = 68 Median (IQR)	
FBS (mg/dl)	164 (120–203)	137 (118–194)	178 (122–213)	0.0384^*^
TC (mg/dl)	162 (143–183)	162 (147–183)	158 (138–183)	0.5117
TG (mg/dl)	157 (113–193)	163 (107–188)	151 (115–200)	0.8141
LDL (mg/dl)	97 (78–115)	98 (82–115)	96.5 (74–115)	0.6538
HDL (mg/dl)	34 (29–38)	35 (31.5–40.5)	33 (27-37.8)	0.03^*^
HbA1C	8.2 (7.4–9.9)	7.8 (7.2–8.85)	8.6 (7.5–10.6)	0.0404^*^
TSH (µIU/ml)	1.82 (1.35–2.73)	1.65 (1.28–2.54)	2.04 (1.38–2.95)	0.0562
SERUM OC (ng/ml)	3.68 (2.67–5.51)*	4.46 (2.76–7.82)	3.39 (2.44–4.42)	0.0135^*^

FBS- Fasting blood sugar; TC- Total cholesterol; TG- Triglycerides; LDL- Low-density lipoprotein; HDL- high-density lipoprotein; TSH- Thyroid-stimulating hormone. * Represents statistical significance at the level of 5%

CC-IMT was measured in all the subjects, and they were divided into two groups i.e., those with CC-IMT ≥ 0.9 mm and those with CC-IMT < 0.9 mm. The total subjects with CC-IMT ≥ 0.9 mm were 60% (n = 68) and those with CC-IMT < 0.9 mm were 40% (n = 45) (Table 1). There was no significant difference in the age, gender distribution smoking status, and duration of diabetics between the two groups (Table 1). Comparison of different biochemical parameters showed significant difference (P < 0.05) in FBS, HbA1c, HDL, and serum OC levels between the two CC-IMT groups (Table 2). There were no statistically significant differences in the lipid parameters including TG, TC, LDL.

We found that the mean HbA1C levels in subjects with CC-IMT ≥ 0.9 mm were higher when compared to those with CC-IMT < 0.9 mm(p-value < 0.05).

### Serum OC and CC-IMT

**Fig. 1:**
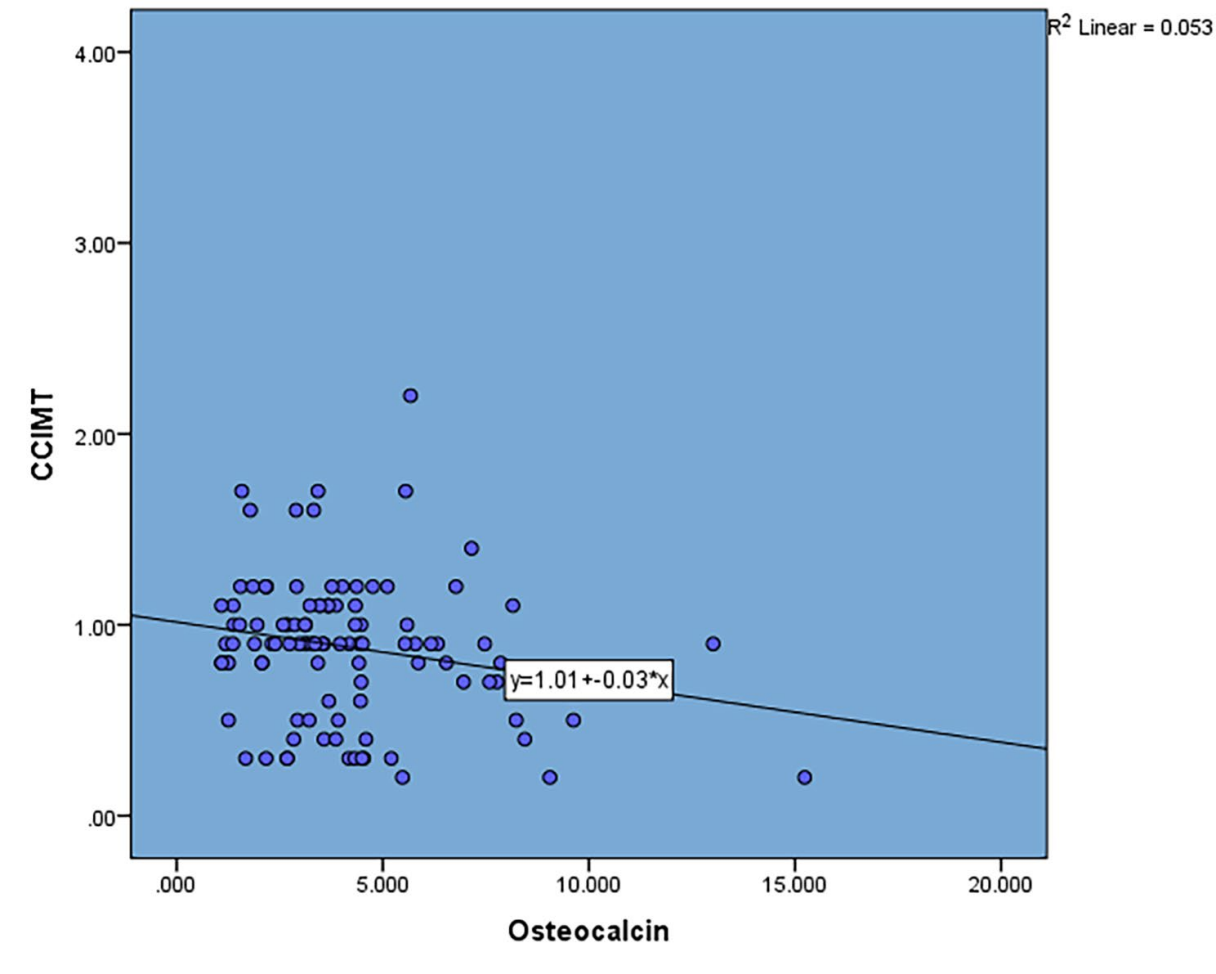
Correlation of CC-IMT with serum osteocalcin levels. Scatter plot showing negative correlation between CC-IMT and serum osteocalcin. Linear regression line is represented with R^2^ value value

**Fig. 2:**
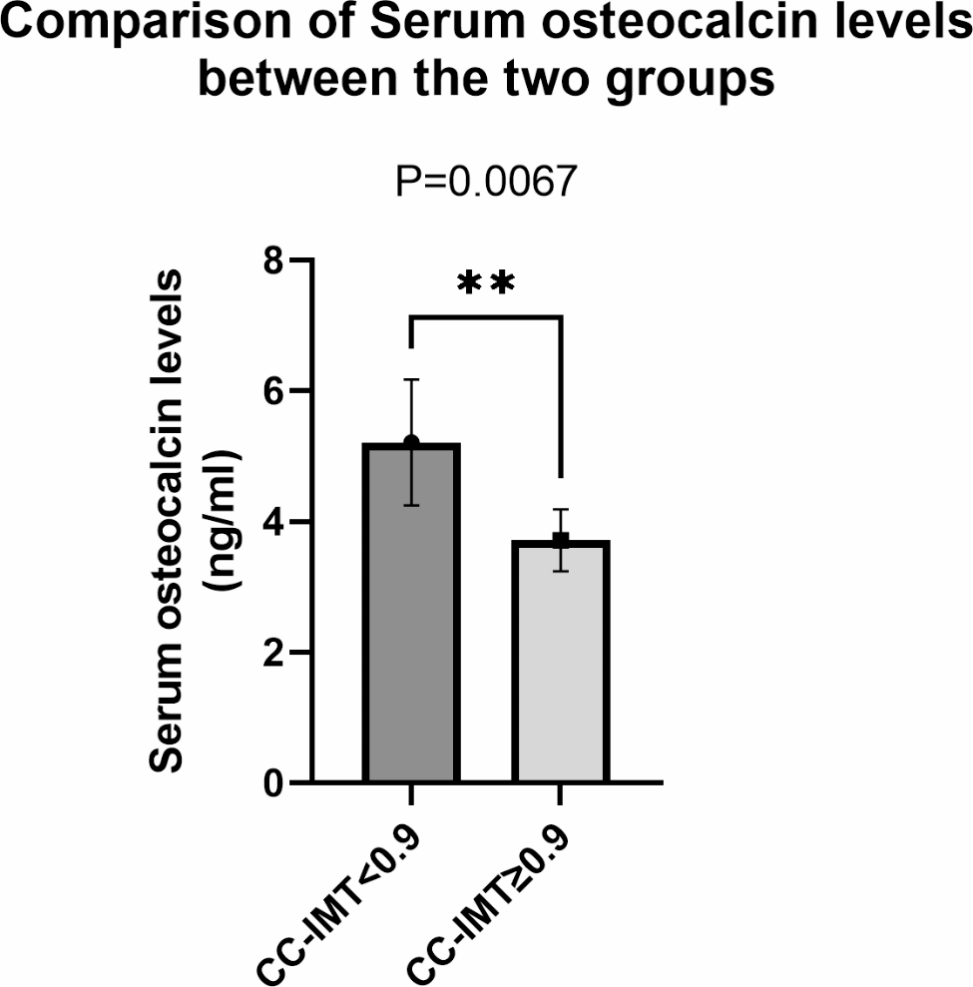
Comparison of serum osteocalcin levels. Box plot with bars showing mean serum OC levels between subjects with CC-IMT ≥ 0.9 mm and those with CC-IMT < 0.9 mm. the error bars represent standard error mean. **, represents statistical significance with a p-value < 0.01

In our study, we found a negative correlation was found between CC-IMT and serum OC levels (r= -0.23, p-value < 0.05) (Fig. 1). Patients with lower levels of CC-IMT had significantly higher levels of serum OC. Median Serum OC level was significantly higher in the patient group with CC-IMT < 0.9. (Fig. 2).

### Serum OC and macrovascular complications (MV)

**Fig. 3:**
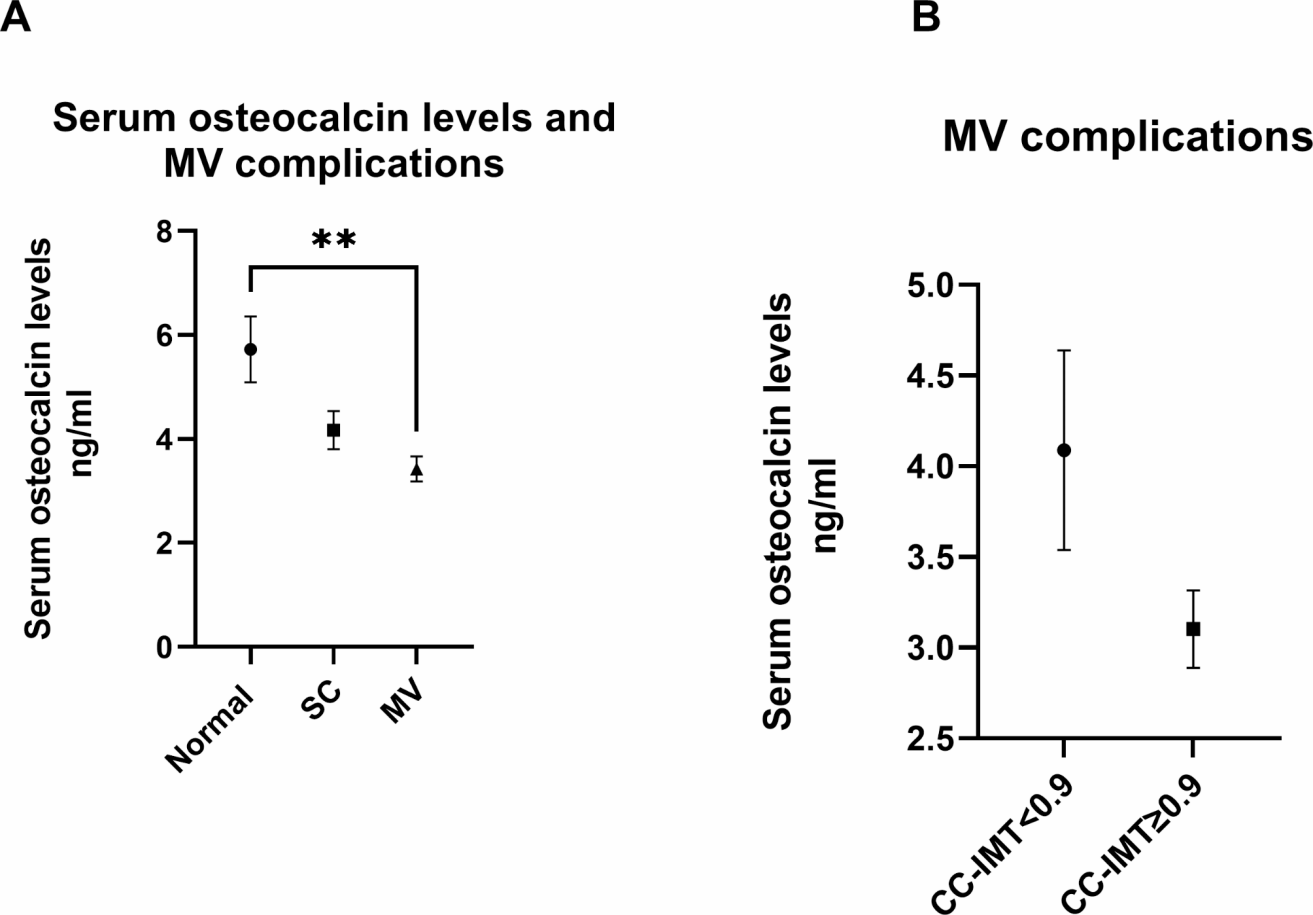
Association of serum OC levels with macrovascular complications. **A.** Mean serum OC levels in different groups with macrovascular (MV) or subclinical (SC) complications. **B** Serum OC and MV complications in groups with CC-IMT < 0.9 and CC-IMT ≥ 0.9. Error bars represent standard error mean. **, represents statistical significance with a p-value < 0.01

Table 3 provides the proportion of patients with MV complications along with their mean serum OC levels. We found that subjects with MV complications had significantly lower (P = 0.0011) OC levels when compared to those without any MV. The mean OC levels in patients with MV is 4.9 ± 3 ng/ml. Among those with MV, 44% had CC-IMT < 0.9. Comparing the serum OC levels in the patients with MV, the level was lower in the CC-IMT ≥ 0.9 than the CC-IMT < 0.9 group (Fig. 3).

**Table 3:** Proportion of patients with macrovascular complications

Group	N	Serum OC levels in ng/ml	p-value
Normal – CC-IMT < 0.9 without MV	31	5.72 ± 3.53	P < 0.0001
SC- CCIMT ≥ 0.9 and without MV	39	4.17 ± 2.3	
MV in Total population	43	3.42 ± 1.56	
MV in CC-IMT < 0.9 mm group	14	4.09 ± 2.06	
MV in CC-IMT ≥ 0.9 mm group	29	3.1 ± 1.15	

MV- Macrovascular complications; SC- Subclinical complications

## Discussion

Our study has tried to establish a correlation between OC levels and atherosclerosis estimated by CC-IMT in T2DM patients. We have used CC-IMT as a measure for atherosclerosis. Studies have shown a strong correlation of increased CC-IMT with risk factors of atherosclerosis. For this, we used the cut-off value of 0.9 and categorized the groups into CC-IMT < 0.9 and CC-IMT ≥ 0.9. However, a small proportion of the population with CC-IMT less than 0.9 may be associated with macrovascular complications due to several other risk factors. So CC-IMT < 0.9 mm and no complications suggest no atherosclerosis.

Several studies have evaluated age as a risk factor for developing atherosclerosis. They have observed a positive correlation between age and CC-IMT showing that as age increases CC-IMT increases [16]. Our study showed a positive correlation between age and CC-IMT however of no statistical significance (r = 0.15, p = 0.09).

A large Japanese study done in 2013 on 370 patients with T2DM showed a direct correlation between CC-IMT and HbA1c (p = 0.0007) [17]. However, another smaller study in Türkiye concluded that there was no significant association between CC-IMT and HbA1c in T2DM patients with atherosclerosis [18]. Our study showed that patients with high CC-IMT had a higher mean HbA1C level of 8.95 ± 1.73 when compared to those with lower CC-IMT (8.35 ± 1.52) (p < 0.05). Hence the subjects with higher HbA1C i.e., with more uncontrolled sugars were associated with a higher CC-IMT level.

A protective association was noted between CC-IMT and HDL in a study among menopausal women in Pittsburg, USA [19]. Another study from Türkiye showed a significant correlation between LDL/HDL and CC-IMT [20]. Our study also showed that higher mean HDL was associated with significantly lower CC-IMT levels indicating a protective effect of HDL on atherosclerosis. Mean HDL levels were higher (35.73 ± 7.42) in the group with CC-IMT < 0.9 mm when compared to the group with CC-IMT < 0.9 mm (32.22 ± 7.90) (p < 0.05). No significant correlation was found between CC-IMT and TC, TG, LDL. Following this, we evaluated the association of serum OC levels with CC-IMT.

### Serum OC and atherosclerosis

Several studies have been done where OC has been compared with different indicators for atherosclerosis like CC-IMT, aortic calcification, coronary angiography [12]. Negative correlation of CC-IMT with serum OC has been observed in middle-aged Chinese individuals [21], post-menopausal women 1 [22, 23]. Sheng et al. compared serum OC and CC-IMT in 817 diabetic individuals and found a negative correlation between OC CC-IMT (r = − 0.110, P = 0.005). They also found that decreased OC levels indicated a higher risk for carotid atherosclerotic plaques (odds ratio of 1.77 for 1 SD decrease in OC, 95% CI 1.23–2.76, p = 0.005) [24]. However, studies done by Reyes-Garcia et al. observed that subjects with higher CC-IMT and the presence of carotid plaques had higher levels of OC levels (2.17 ± 1.84ng/ml) compared to (1.25 ± 0.67 ng/mL) among patients without carotid plaques (*P* = 0.042) [11, 25]. This finding is interestingly contrary to the findings in our study. This discordant data may be because the relationship between OC and glucose is influenced by the presence of diabetes, diabetes progression and the severity of atherosclerosis. Our study showed a significant negative correlation between serum OC and CC-IMT (r= -0.23, p value < 0.05), so the subjects with higher OC had lower CC-IMT levels. This could point to a protective effect serum OC may have in atherosclerosis, however further studies are needed to confirm its role. Regression analysis was performed to see the impact of demographic characteristics and biochemical parameters on atherosclerosis development. We found that CC-IMT, HbA1c, and osteocalcin strongly correlated.

### Serum OC levels and macrovascular complications

The consequence of atherosclerosis is the presence of MV events. Hence, we analyzed the association of OC levels with MV complications. In our study, patients with MV complications were found to have a significantly lower OC level compared to the population without MV complications. A similar association has been observed by other studies. Bao et al. [26] showed that serum OC decreased significantly in patients with coronary artery disease assessed by a coronary angiography when compared with those without CAD (P = 0.029). Patients with abnormal coronary angiography had significantly lower serum OC levels compared to normal angiography (P < 0.001). These conclusions were similar to our study and showed the protective role of osteocalcin.

Though there were no studies found comparing serum OC levels to OC levels in atherosclerotic plaques, there is evidence to note that Osteocalcin is present in calcified aortic tissue and heart valves, and either not detectable or present at very low levels in non-mineralized lesions and normal tissue. A study by Levy et al. compared the OC levels in calcified plaque (50.9 ng/mg) and fibrous plaques (1.1 ng/mg) and concluded that OC levels are significantly elevated in calcified atheromatous plaques in comparison to non-calcified plaques [27].

### Strengths and limitations

Few studies addressed this link between OC and atherosclerosis, especially in the Indian population, which has a higher cardiovascular risk for diabetes, compared to other ethnicities. The study was done measuring CC-IMT, which is a reasonably reliable marker of the state of atherosclerosis all over the body. These are the strengths of this study.

The study is cross-sectional, and there was no follow-up of different groups of patients, to assess if vascular events indeed were more common in patients with low OC. The sample size may have to be larger, to substantiate some of the findings of this study.

## Conclusion

Our study found that serum OC was lower in subjects with higher CC-IMT showing a significant negative association between atherosclerosis and serum OC levels in T2DM patients. This points to a protective role of OC in atherosclerosis where a high level of OC may be associated with lower risks of developing atherosclerosis. This association further extended to the severity of atherosclerosis, where patients with macrovascular complications were found to have a significantly lower OC level. From our study, serum OC can be considered a novel diagnostic marker for atherosclerosis. In the future, it could be a therapeutic target to ameliorate vascular complications in high-risk T2DM individuals.

## Data Availability

The datasets used and/or analysed during the current study available from the corresponding author on reasonable request.
