# Correction: Association between serum osteocalcin and atherosclerosis in type-2 diabetes mellitus: a cross-sectional study

**DOI:** 10.1186/s12902-024-01538-z

**Published:** 2024-01-19

**Authors:** Vishal Chandra Sharma, Sudha Vidyasagar, Cynthia Amrutha Sukumar, Nanda Krishna B, Sharanya Shree

**Affiliations:** 1 Department of Medicine, Kasturba Medical College, Manipal, Manipal Academy of Higher Education, Manipal, India; 2 Department of Medicine, Trinity Health, Oakland, CA USA

**Correction:** ***BMC Endocr Disord*** **23, 269 (2023)**

10.1186/s12902-023-01462-8

Following publication of the original article [1], the authors reported that the abbreviation “KMC” should be “Kasturba Medical College” in affiliation 1.

The correct affiliation 1 should read: Department of Medicine, Kasturba Medical College, Manipal, Manipal Academy of Higher Education, Manipal, India.

The original article [1] has been updated.
